# 

**Published:** 1857-12

**Authors:** 


### INDEX TO VOL. VI.

PAGE.

Alcoholic Liquors, Effects of, in Phthisis, by N. S. Davis, M. D. .... 48
American Medical Association, Transactions of, for 1856 .... 178, 290, 90, 52
Annual Address to the Esculapian Medical Society, by H. R. Payne, M. D. .... 55
American Pharmaceutical Association, Proceedings of, 1856 .... 79
Abstract of Medical Sciences, Half-Yearly ... 138
Anniversary Address, by Isaac Ferris, D. D., L. L. D., etc., etc., 1857 .... 138
Address on the Life and Character of R. M. Porter, M. D., by J. B. Lindsly, M. D. .... 139
Alcohol, Effects of, on the Diseases of Europeans in India .... 143
Academy of Natural Sciences of Chicago ... 144
Amylene, a new Anæsthetic .... 149
American Med. Associ'n, Delegates to ... 191, 188
Agent for the Journal .... 189
Alcohol, Influence of, on Tuberculosis .... 190
Address to DeWitt Co. Medical Society, by Christopher Goodbrake, M. D .... 247
American Medical Association at Nashville, May, 1857 .... 332
Advertisement .... 339
Abortion Case, Fatal Result .... 386
A New Pathy .... 432
Ammonia, Valerianate of, by W. Hay, M.D ... 444
An Amputation in Paris, Report of, by J. C. Morfit, M. D. .... 494
Aconite, Use of, as a Therapeutic Agent, by E. B. Stevens, M. D. .... 510

Bursæ, Enlarged, Treatment of, by J. B. Prowse, M.D .... 506
Blepharospasm and Photophobia relieved by Chloroform .... 513
Body Snatching — Resurrectionists — The Remedy. By the Editor .... 525
Biographical Sketch of Dr. Marshall Hall ... 470
Blood, Changes of, in Continued Fever, by the Editor .... 391
Biennial Report of the Board of Trustees of the Ill. State Hospital for the Insane .... 79

Correspondence, Editorial, of the Atlanta Medical Journal .... 166, 39
Chemistry, Introduction to Practical, by J. E. Bowman, M. D .... 45
Chlorate of Potassa in Typhoid Fever, by E. C. Elliot, M. D .... 112
Chloroform, on the general use of, by Dr McLeod .... 119
Clinical Cases treated in the Surgical Wards of Mercy Hospital, by J. W. Freer, M. D .... 163
Controversy, Medical, by the Editor .... 190
Correspondents .... 292, 196
Change, Editorial .... 341, 197
Chloroformization in Midwifery, by J. N. Graham, M. D .... 199
Cases occurring in the Practice of Prof. D. Brainard, reported by E. Powell, M. D .... 214
Cook Co. Medical Society, Proceedings of .... 451, 315, 29[illegible], 237

PAGE.

Clinical Lecture on Ulceration of the Cornea, etc., by the Editor .... 262
Clinical Lectures on Diseases of the Urinary Organs, by R. B. Todd, M. D .... 284
Congenital Non-Secretion of Urine, by Thos. Hall, M. D .... 346
City Hospital and Board of Health .... 383
Changes, Professional .... 388
Cases in Practice, by E. K. Bowman, M. D ... 409
Crawford the Sculptor and Prof. Gibson .... 424
Communication, by S. Ramsey, M. D .... 449
Clinical Instruction .... 479
Cholera, Epidemic .... 480
Climate, its Effects on Tubercular Affections, by Edwin Lee, M. D .... 519
Cases of Cancer Treated at the College Clinic by Prof. Brainard, rep'd by E. Powell, M.D. 552
Chloroform in Paroxysmal Typhoid Fever, by F. W. White, M.D .... 559
Close of the Volume .... 578

Dislocation of the Long Bones, etc., by F. H. Hamilton, M. D .... 523
Death of Dr. Marshall Hall .... 435
Dysentery — Mortality of Chicago, by the Editor .... 384
Death of Dr. Thomas Spencer .... 340
DeWitt County Medical Society, Proceedings of .... 432, 95, 241
Death of Dr. Kane .... 186
Death of Dr. Paris .... 99
Death from Air in the Veins after Parturition. 516

Electricity in the Suppression of the Lacteal Secretion .... 516
Epilepsy, by Edward Brown-Sequard, M. D., 452, 418, 378, 272, 215, 175, 127
Extensive Injury of the Upper Extremity, by Thos. Hall, M. D .... 322
Esculapian Society, Proceedings of .... 355
Education, Medical .... 429
Epilepsy, Ligaturing of Common Carotid Artery, by C. Angel, M. D .... 446
Empyemia, Treated by Injections of Iodine, by D. Brainard, M. D .... 487
Esculapian Society, Annual Meeting of .... 570

Female Diseases, Rigby on the Constitutional Treatment of .... 185
Fœtus at Full Period without Membranes, etc., by J. P. H. Stevenson, M. D .... 209
Fayette County Medical Society .... 337
Fiske Medical Prize Questions .... 426
Fractures of the Scapula, Remarks on, by L. A. Dugas, M. D .... 428

Graafian Vesicle, Impregnation of, by G. S. Dewey, M. D .... 352
Glycerine in Phthisis, by the Editor .... 97

Hæmoptysis, as it prevailed in Little Rock, by C. M. Almont, M. D .... 343
Hæmoptysis, as a sign of Pulmonary Phthisis, by E. DeLamare, M. D .... 220

Heart, Valvular Disease of, etc., by F. R. Payne, M. D....159
Homœopathic Medical Preparations, Analysis of, by E. H. Parker, M. D.... 33
Hydrophobia....481

Introductory Lecture, by E. D. Fenner, M.D., 78
Introductory Lecture, by Alfred Stille, M.D., 78
Introductory Lecture, by Chandler R. Gilman, M. D.... 78
Illinois State Medical Society, Annual Meeting of....244, 189
Iodide of Zinc as a Local Application....196
Iodine for Vomiting in Pregnancy....197
Iodine in Scrofula, Phthisis, Pulmonalis, etc. by Benj. Woodward, M. D....257
Illinois State Medical Society....433, 336
Indiana State Med. Society, Transactions of, 364
Introductory Address, by the Editor....535

Knee Joint, Injuries of, by Benj. Woodward, M.D....162

Milk-Sickness, Pathology of, by W. H. Phillips, M. D.... 7
Medical World.... 47
Medical Profession and Scientific Observers, 52
Mortality of Chicago in 1856, by the Editor, 98
Medical Journals.... 99
Mercury in Typhoid Fever, by W. M. Chambers, M. D....147
Milk-Sickness, Etiology of, by G. W. Wilkinson, M.D....151
Milk, Dispersion of....198
Mortality of Women in Childbirth....198
Medical Notes and Reflections, by Sir Henry Holland, M. D....229
Milk-Sickness, Pathology of, in Parke Co., by John Pickard, M.D....260
Medical Schools and Medical Politics....340
Malignant Disease, a Case of, by N. O. Pearson, M. D....350
Medical Education, by the Editor....479
Milk-Sickness, Twenty Propositions on, by J. W. Crooks, M.D....489
Milk-Sickness, by J. C. Beck, M.D....496
Mamma, Treatment of, during Childbed, by Dr. Durr....499
Midwifery Practice, Sketches in, by Walter Channing, M. D....502
Medical Student's Vade Mecum, by G. Mendenhall, M. D....576

New Castle Medical Society....574

Ovarian Cysts....497
Ottawa Medical Society....535
Ovariotomy, a Successful Case of, by S. Long, M.D....103
Obstetrics—the Science and the Art.... 44
Ovariotomy, History and Statistics of.... 46

Personal....524
Puerperal Fever, Causes, etc....521
Pregnancy, its Influence on Tuberculosis...519
Practical Anatomist, by J. M. Allen, M. D... 44
Prescription Book.... 45
Prolapsus of the Vagina, by L. C. White, M.D., 76
Prurigo, by the Editor.... 98
Puerperal Fever, Preventive Treatment....221
Paralysis, a Clinical Lecture on....222
Pregnancy, Signs and Symptoms of....323
Placenta Retained, by F. K. Bailey, M D....348
Physiology, Manual of....427
Principles of Medicine....427
Physiological Anatomy and Physiology of Man....462
Pulmonary Consumption, Diagnosis of, by the Microscope....507
Pleura above the Clavicle....512
Pneumonia, Bloodletting in....513
Paralysis, a Case of, by C. Brackett, M.D....448
Practical Receipts for Everyday Use, the Handbook of, by T. F. Branston....576

Quinine in Fever, by J. Casselbury, M.D....428

Report of Surgical Cases, etc., by Geo. K. Ammerman, M. D....439
Rush Medical College....338, 478, 578
Rock Island Medical Society....413
Report of Representatives of Rock Island Medical Society....414
Report of Committee on Practical Medicine, by F. R. Payne, M. D....295
Report on Meteorological Changes, etc., in Chicago, by the Editor.... 12
Report of Case Involving Loss of Corresponding Portions of both Hemispheres of the Brain, by L. N. Dimmick, M.D....557
Remarkable Cases in Surgery, a Collection of, by P. F. Eve....577

Scalp Wound, followed by Arachnitis, etc., by Geo. Ammerman, M.D.... 68
Strychnine, is it Cumulative in the System, 125
State of N. York Med. Society Transactions, 232
Subscribers....478, 338
Scorbutus—Scurvy, by Thos. A. Hill, M. D., 354
S. Carolina Med. Association Transactions of, 468

Tubercular Tendency, can it be Eradicated, by Benj. Woodward, M. D....404
Translations by Dr. Byford—
Death Caused by Hemorrhage from the Vaginal Mucous Membrane....564
Abortive Treatment of Extrauterine Fœtation, by Dr. Burci....565
Treatment of Phthisis Pulmonalis....566
Congenital Idiopathic Cerebro-Spinal Meningitis, by M. Stoltz....567

Union Med. Assoc'n, Annual Meeting of, 528, 360
Ununited Fractures, by C. Brackett, M. D...548

Veratrum Viride as a Poison, etc., by J. S. Pashley....494
Veratrum Viride, Use of, by Dr. Hill....211
Veratrum Viride in Fevers, by A.W. Dewey, M.D....110
Veratrum Viride, Tincture of.... 97
Valvular Aortic, Constrictive Disease, by F. B. Norcom, M. D....108

Water Externally Applied, Effects on the Circulation....512

Yellow Fever, Cause of.... 86

Death Caused by Hemorrhage from the Vaginal Mucous Membrane... 564

Abortive Treatment of Extrauterine Fœtation by Means of Electricity. By Dr. Burci of Pisa 565

Treatment of Phthisis Pulmonalis 566

Congenital Idiopathic Cerebro-Spinal Meningitis. By M. Stoltz 567

PROCEEDINGS OF MEDICAL SOCIETIES—

Annual Meeting of the Esculapian Society 570

New Castle (Ind.) Medical Society 574

BOOK NOTICES—

The Medical Student's Vade Mecum. By G. Mendenhall, M.D 576

The Handbook of Practical Receipts for every day use. By T. F. Branston. 576

A Collection of Remarkable Cases of Surgery. By P. F. Eve. 577

EDITORIAL—

Close of the Volume 578

Rush Medical College Clinic 578

### RECEIPTS ON SUBSCRIPTIONS,

UP TO DEC. 20TH, 1857.

### ILLINOIS.

| | | |
|---|---|---|
| Dr. E. C. Ellett | VOL. 1, 1858 | $2 00 |
| " H. C. Donaldson | VOLS. 6 & 1, 1857-8 | 4 00 |
| " N. Halton | VOL. 6, 1857 | 2 00 |
| " Vageler | " 6, " | 2 00 |
| " M. Wiley | " 6, " | 2 00 |
| " A. E Goodwin | " 6, " | 2 00 |
| " Latta Wallace | " 6, " | 2 00 |
| " J. M. Steele | " 1, 1858 | 2 00 |
| " D. W. Stormont | " 1, " | 2 00 |
| " L. N. Dimmick | " 1, " | 2 00 |

### INDIANA.

| | | |
|---|---|---|
| Dr. Charles D. Pearson | VOL. 6, 1857 | $2 00 |
| " H. A. McFarland | " 1, 1858 | 2 00 |
| " R. C. Black | " 1, " | 2 00 |

### MICHIGAN.

| | | |
|---|---|---|
| Dr. M. B. Beers | VOLS. 4, 5 & 6 | $6 00 |

### WISCONSIN.

| | | |
|---|---|---|
| Dr. A. B. Carey | VOL. 6, 1857 | $2 00 |
| " M. Waterhouse | " 1, 1858 | 1 00 |
| " J. Phillips | " 6, 1857 | 2 00 |
| " C. Marsh | " 6, " | 2 00 |

### IOWA.

| | | |
|---|---|---|
| Dr. J. H. Griffith | VOL. 6, 1857 | $2 00 |
| " J. W. Cowden | " 1, 1858 | 2 00 |
| " J. Williamson | " 6, 1857 | 2 00 |
| " H. Love | " 6, " | 2 00 |

### MISSOURI.

| | | |
|---|---|---|
| Dr. Wm. G. Elder | VOLS. 5, 6 & 1 | $5 00 |

### ALABAMA.

| | | |
|---|---|---|
| Dr. C. McDonough | VOL. 1, 1858 | $2 00 |

